# Image-based classification of wheat spikes by glume pubescence using convolutional neural networks

**DOI:** 10.3389/fpls.2023.1336192

**Published:** 2024-01-12

**Authors:** Nikita V. Artemenko, Mikhail A. Genaev, Rostislav UI. Epifanov, Evgeny G. Komyshev, Yulia V. Kruchinina, Vasiliy S. Koval, Nikolay P. Goncharov, Dmitry A. Afonnikov

**Affiliations:** ^1^ Institute of Cytology and Genetics of the Siberian Branch of the Russian Academy of Sciences, Novosibirsk, Russia; ^2^ Department of Mathematics and Mechanics, Novosibirsk State University, Novosibirsk, Russia; ^3^ Kurchatov Center for Genome Research, Institute of Cytology and Genetics of the Siberian Branch of the Russian Academy of Sciences, Novosibirsk, Russia

**Keywords:** wheat, wheat species, pubescent (hairy) glume, image analysis, segmentation, deep learning, convolutional neural network, phenotyping

## Abstract

**Introduction:**

Pubescence is an important phenotypic trait observed in both vegetative and generative plant organs. Pubescent plants demonstrate increased resistance to various environmental stresses such as drought, low temperatures, and pests. It serves as a significant morphological marker and aids in selecting stress-resistant cultivars, particularly in wheat. In wheat, pubescence is visible on leaves, leaf sheath, glumes and nodes. Regarding glumes, the presence of pubescence plays a pivotal role in its classification. It supplements other spike characteristics, aiding in distinguishing between different varieties within the wheat species. The determination of pubescence typically involves visual analysis by an expert. However, methods without the use of binocular loupe tend to be subjective, while employing additional equipment is labor-intensive. This paper proposes an integrated approach to determine glume pubescence presence in spike images captured under laboratory conditions using a digital camera and convolutional neural networks.

**Methods:**

Initially, image segmentation is conducted to extract the contour of the spike body, followed by cropping of the spike images to an equal size. These images are then classified based on glume pubescence (pubescent/glabrous) using various convolutional neural network architectures (Resnet-18, EfficientNet-B0, and EfficientNet-B1). The networks were trained and tested on a dataset comprising 9,719 spike images.

**Results:**

For segmentation, the U-Net model with EfficientNet-B1 encoder was chosen, achieving the segmentation accuracy IoU = 0.947 for the spike body and 0.777 for awns. The classification model for glume pubescence with the highest performance utilized the EfficientNet-B1 architecture. On the test sample, the model exhibited prediction accuracy parameters of F1 = 0.85 and AUC = 0.96, while on the holdout sample it showed F1 = 0.84 and AUC = 0.89. Additionally, the study investigated the relationship between image scale, artificial distortions, and model prediction performance, revealing that higher magnification and smaller distortions yielded a more accurate prediction of glume pubescence.

## 1 Introduction

### 1.1 Glume pubescence in wheat

Pubescence, an important phenotypic trait in vegetative and generative plant organs (Johnson, 1975; Karabourniotis et al., 2020; Shvachko et al., 2020), provides increased resistance to drought, pests, and various environmental factors (Fahmy, 1997; Bickford, 2016; Moles et al., 2020). This trait serves as a crucial morphological marker and is actively studied in genetic and breeding research aimed at developing crop varieties with enhanced resistance to stressful environmental factors (Papp et al., 1992; Du et al., 2009; Doroshkov et al., 2014).

Wheat is one of the most significant crops thriving in a wide ecological range (Volis et al., 2015; Sadras, 2021), exhibiting pubescence as an important adaptive trait on leaves, leaf sheath, glumes, and nodes (Dobrovolskaya et al., 2007). Similar to leaf pubescence (Pshenichnikova et al., 2019), glume pubescence in wheat is linked to the plant’s adaptive properties seemingly contributing favorably to drought or cold tolerance (Threthowan et al., 1998)—for instance, analysis of floret temperatures in freezing conditions indicated higher temperatures in pubescent florets compared to their glabrous counterparts (Maes et al., 2001). Studies of landrace populations of tetraploid wheat collected at various altitudes in Ethiopia revealed that up to 20% of accessions above 2,300 m have pubescent glumes, while glume hairiness was completely absent in accessions from lower altitudes (Eticha et al., 2005). However, studies on bread wheat cultivars and crosses with pubescent and glabrous glumes indicated similar agronomic traits, showing no significant influence on productivity in these accessions governed by the genes controlling glume pubescence (McNeal et al., 1971). Glume pubescence emerges as an important trait in wheat genetic studies (Tsunewaki, 1966; Khlestkina et al., 2006; Luo et al., 2016; Hu and Zuo, 2022), demonstrating linkage to several important genes/loci such as resistance to *Blumeria graminis* (DC) Speer f. sp. *tritici* Marchal (syn. *Erysiphe graminis* DC f. sp. *tritici* Marchal) (Briggle and Sears, 1966) and spikelet size and number (Echeverry-Solarte et al., 2015) among others (Hu and Zuo, 2022). Furthermore, glume pubescence serves as a classification trait, adding in the determination of wheat species and varieties (Goncharov, 2011; Yen et al., 2020)—for example, in *Triticum timopheevii* (Zhuk.) Zhuk. and *T. vavilovii* Jakubz., all accessions have haired glumes (Goncharov, 2011).

### 1.2 Image analysis for plant pubescence evaluation

Determining pubescence and assessing its characteristics usually necessitate a visual analysis of plants conducted by an expert. Methods devoid of a binocular loupe tend to be subjective. Recent developments include automatic techniques for analyzing pubescence primarily in leaves. These methods are based on analyzing 2D images obtained via a microscope (Genaev et al., 2012; Pomeranz et al., 2013; Mirnezami et al., 2020) or 3D images (Kaminuma et al., 2008; Bensch et al., 2009; Failmezger et al., 2013). They not only identify trichomes on plant organs but also estimate their number and size, employing machine vision algorithms and demonstrating high accuracy and swift data processing. This demonstrates the potential of using image analysis to assess pubescence characteristics across various plant organs.

### 1.3 Deep machine learning for plant image analysis

Remarkable strides in image analysis has been achieved through the utilization of deep machine learning neural networks. Deep learning networks are characterized by a multilayered architecture where subsequent layers utilize the output of the previous layer as input to extract features related to the analyzed objects (Ubbens and Stavness, 2017; Singh et al., 2018; Demidchik et al., 2020; Alzubaidi et al., 2021; Xiong et al., 2021). These approaches enable the automatic extraction of image features with regression or classification in a single pipeline, trained simultaneously from end to end (LeCun et al., 2015).

Deep learning algorithms are categorized based on how input data is prepared (Alzubaidi et al., 2021; Wang et al., 2021; Yan and Wang, 2022): supervised learning, semi-supervised learning, and unsupervised learning. Supervised learning demands all input data to be expert-labeled, requiring an image training dataset to derive network parameters. This technique, popular in solving plant phenotyping image analysis tasks (Ubbens and Stavness, 2017; Singh et al., 2018), can tackle image segmentation, classification, and object detection (Jiang and Li, 2020; Sanaeifar et al., 2023).

Supervised learning has found success in plant phenomics for disease recognition (Barbedo, 2019; Liu and Wang, 2021), plant stress detection (Azimi et al., 2021), morphometrics (Kurbanov et al., 2020; Gibbs et al., 2021), weed detection (Hasan et al., 2021; Rai et al., 2023), and plant classification into different species (Dyrmann et al., 2016; Sundara Sobitha Raj and Vajravelu, 2019; Liu et al., 2022). However, it requires large labeled datasets, involving substantial expert effort in preparation and labeling (Minervini et al., 2016; Barbedo, 2018). Consequently, semi-supervised and unsupervised learning methods have gained recent traction (Yan and Wang, 2022).

Semi-supervised learning involves training samples with labeled and unlabeled images, where unlabeled images can receive pseudo-labels or be assigned negative labels based on trained networks. This approach has been utilized in plant disease analysis (Zhou et al., 2023), counting cotton balls (Adke et al., 2022), and plant shoot counting (Karami et al., 2020).

Contrarily, unsupervised learning techniques for image analysis do not rely on labeled datasets and are akin to solving clustering problems without known object classes (Alzubaidi et al., 2021; Yan and Wang, 2022). These methods have been employed for tasks such as image denoising, reconstruction, generation, clustering, and dimensionality reduction (Raza and Singh, 2021; Akçakaya et al., 2022). They have found applications in plant phenomics, including plant image generation (Madsen et al., 2019), disease recognition (Nazki et al., 2020; Benfenati et al., 2023), leaf segmentation (Al-Shakarji et al., 2017), weed recognition (Hu et al., 2021), and plant development prediction (Drees et al., 2021). Some studies propose modifying neural network structures to transform original unsupervised problems into supervised ones using predefined kernels and only patches from the input test image (Yan et al., 2024).

In supervised learning problems for image analysis, various deep convolutional neural network (CNN) architectures have gained immense popularity (Toda and Okura, 2019; Jiang and Li, 2020). CNNs encompass convolutional layers—sets of repetitive image filters convoluted to images or feature maps—alongside pooling layers. These networks interpret images by converting them into numerical values, successfully addressing plant image analysis tasks such as segmentation, object detection, and classification (Duong et al., 2020; Saleem et al., 2020; Seki and Toda, 2022).

### 1.4 Related works

This paper proposes a method to classify wheat spikes based on their digital images according to the presence or absence of glume pubescence. Notably, no previous work has specifically addressed the pubescence of wheat glumes. However, in a study by Grillo et al. (2017), 138 descriptors of glume shape, size, and color were used for classifying wheat landraces using the linear discriminant analysis (LDA) algorithm. The classification performance was 100% for distinguishing *T. aestivum* L., *T. durum* Desf., and *T. turgidum* L., achieving 100% correct classification for five landraces belonging to *T. aestivum* species and 89.7% for 39 landraces of durum wheat (Grillo et al., 2017).

One of the intriguing tasks in spike image analysis is detecting and counting spikes in field images (Li et al., 2022; Wen et al., 2022; Zhang et al., 2022; Sanaeifar et al., 2023). However, several studies focus on identifying spikes in laboratory conditions or greenhouses, where plants or ears are imaged against a uniform background, facilitating ear identification through segmentation. Bi et al. (2010) utilized 2D images of wheat spikes against a black background to assess various characteristics, such as spike length and awn number and length, and classified the spike shape type according to its length-to-width ratio. Segmentation was performed using the Otsu algorithm. The backpropagation neural network was designed to classify spike images into four wheat varieties using spike morphometric parameters as input. The recognition accuracy rate was 88%.

Qiongyan et al. (2017) implemented a neural network-based method using Laws texture energy for spike detection in the images of plants in the pot obtained in glasshouse conditions. Image segmentation was performed before the spike detection into background and plant regions using five color indices. These indices depended on the R, G, B and hue channel intensities. The performance of the segmentation method was not reported. The accuracy of spike detection varied from 86.6% (single plant in the pot) to 81.5% (four plants in the pot).

Narisetti et al. (2020) modified the algorithm proposed by Qiongyan et al. (2017) by using the wavelet amplitude as an input to the Laws texture energy-based neural network. They also suppressed non-spike structures on the image (leaves and stems) by combining the result of the neural network prediction with a Frangi-filtered image. As a result, the accuracy of spike detection in the images increased to 98.6% on the test dataset.

In the work by Ullah et al. (2021), spikes were detected and counted in images of wheat plants obtained in greenhouses using detection and segmentation algorithms. Several deep learning neural network architectures were tested for ear detection: SSD with Inception resnet v2 as a backbone, Faster-RCNN with Inception v2 as a backbone, YOLOv3 with Darknet53 as a backbone, and YOLOv4 with CSPDarknet53 as a backbone. Networks were trained using 234 images. The performance of spike detection measured as the AP_0.5_ value on the set of 58 test images varied from 0.78 for SSD to 0.95 for Faster-RCNN models. Three network models were used for spike segmentation: shallow artificial neural network (ANN), U-net with a VGG-16 backbone, and DeepLabv3+ with ResNet101 were used for spike segmentation. The Jaccard index (IoU parameter) varied for these methods from 0.610 (ANN) to 0.922 (DeepLabv3+).

The work by (Qiu et al., 2022) is aimed at the problem of spikelet detection on images of spikes against a white background. Before spikelet detection, the images were segmented into spike and background. This procedure was performed using the watershed algorithm. The authors do not provide an estimate of the accuracy of image segmentation but report that it was successfully used to identify candidate spikelets.

A number of approaches were developed for counting spikelets in the images without spike segmentation. In the work by Pound et al., the problem of detecting spikelets in an ear was addressed for wheat images acquired in a greenhouse (Pound et al., 2017). In this work, no spikelet segmentation was performed, but the whole spike region could be distinguished due to closely spaced spikelets on the processed images. Hammers et al. (2023) detected spikelets based on VGG16, the ResNet152V2, and the EfficientNetV2L deep learning models. Shi et al. (2023) detected spikelets in wheat images captured in the field. The authors implemented YOLOv5s-T network model to count spikelets and obtained *R* ^2^ between manual and deep learning counts from 0.85 to 0.97 depending on the plant development stage.

In a previous work, the authors of this paper performed spike segmentation on images acquired in laboratory conditions against a blue background (Genaev et al., 2019). Spike segmentation was performed based on a thresholding algorithm in HSV space. Both the spike body and awns were identified in the image. The Jaccard coefficient (IoU) for spike segmentation was 0.932 and for awns 0.634. The shape and size of the spike body were described by a geometric model. Its parameters made it possible to classify spikes by three types (compact, normal, and spelt) by ML algorithms. The best performance was achieved for the random forest model (F1 = 0.85). The spike geometric parameters were later used to compare the spike shape for hexaploid and tetraploid wheat accessions (Pronozin et al., 2021).

Deep learning was applied to solve tasks related to plant pubescence detection and analysis. It was used to classify soybean crops according to the type of leaf pubescence on multispectral aerial images (Bruce et al., 2021). The authors used a support vector machine classifier with a radial basis function to classify multispectral images of soybean plots into three classes by pubescence. The overall classification accuracy was 83.1%.

Neural networks of various architectures were applied to identify and count trichomes on cotton leaves. Rolland et al. (2022) implemented the deep learning neural network HairNet to classify cotton leaf images obtained using a handheld microscope into nine classes by the intensity of trichome occurrence on their veins and surface. Five ResNet architectures were tested. They demonstrated different performance depending on the accuracy measurement method. ResNet34 demonstrated the best performance (84.85% for accuracy of all images and 91.36% for leaf accuracy). The authors used different dataset stratification strategies (leaf-based splits, year-based splits, and environmental-based splits).

Luo et al. (2023) developed a deep learning approach to detect and quantify trichomes on cotton leaves and stems. The trichomes on the stem edge and leaf edge were photographed using an Olympus szx10 stereoscope with ×12.5 magnification. The authors evaluated three network models: YOLOv3, YOLOv4, and YOLOv5 with different backbones and Mask R-CNN. The work demonstrated that the YOLOv5 network outperformed other YOLO models (F1 = 83% for single trichomes and 97% for clustered trichomes), and Mask R-CNN outperformed YOLOv5 on the images without separation of the single/clustered trichomes dataset (mAP@0.5% 96.18 vs. 74.45).

In the current work, classification approaches were implemented to attribute spike images into classes with haired or hairless glumes. This paper makes the following contributions:

1. The phenotyping of the novel wheat trait, glume pubescence, was proposed on the basis of the spike image classification.
2. The multistep deep learning approach was applied to solve this task. First, image segmentation is performed to detect the spike region; second, spike images are classified by glume pubescence into haired and hairless classes.
3. The deep learning segmentation model outperformed the previous method of spike and awns detection based on machine vision algorithms. The classification model based on the EfficientNet-B1 architecture demonstrated high performance (AUC parameter from 0.86 to 0.96 depending on the dataset stratification method) and robustness with respect to small image distortions in the classification of the whole spike by glume hairiness.

## 2 Materials and methods

### 2.1 Methods overview

**Figure 1:**
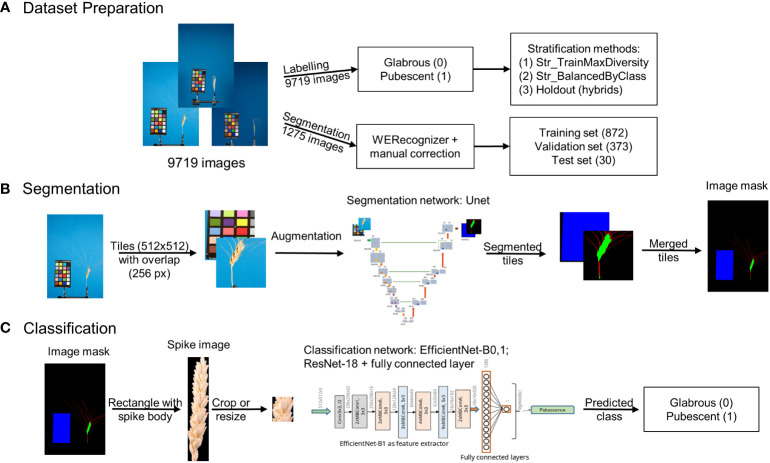
Overview of the integrated approach for analysis of wheat spike images to classify them based on glume pubescence. **(A)** Image dataset preparation, **(B)** image segmentation pipeline, and **(C)** spike image classification pipeline.

An integrated approach using deep machine learning neural networks to classify spike based on glume pubescence relies on images obtained through a standard laboratory protocol. The ear occupies a relatively small area in the image, with the rest being uninformative. Hence, the approach initially involves image segmentation to extract the spike body region from the image, followed by glume pubescence prediction for the spike image fragment. **Figure 1** summarizes the methods employed in this work.

The analysis included three steps: preparation of image datasets to train and test neural networks for segmentation and classification tasks (**Figure 1A**), development of an image segmentation method for identification of the spike region (**Figure 1B**), development of a method for the classification of spike body images by the type of glume pubescence (pubescent or glabrous) (**Figure 1C**). Special algorithms were developed for dataset preparation and stratification, taking into account wheat species diversity and the proportion of spikes of the studied accessions with pubescent/glabrous glumes. In developing deep machine learning methods, various neural network topologies were investigated.

### 2.2 Biological material and spike imaging

Material from the collection of N.P. Goncharov (Institute of Cytology and Genetics of the Siberian Branch of the Russian Academy of Sciences) was used in the study. It included 239 accessions of 19 wheat species. Their list is given in **Supplementary Table S1**. Wheat accessions included species of different ploidy (three diploid, eight tetraploid, and seven hexaploid). Hybrids of different wheat species were also used for analysis (see **Supplementary Table S2**). The analysis was performed for the main spike of the plants.

**Figure 2:**
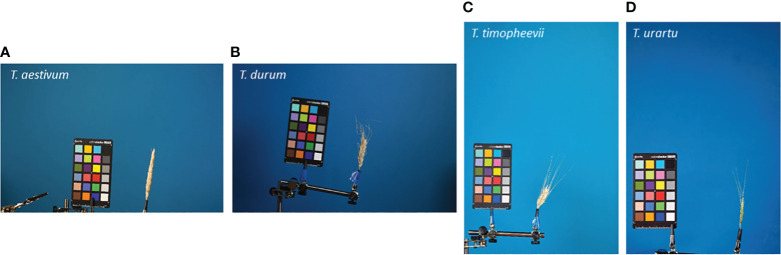
Examples of the spike images obtained using the “clip” protocol. The species names are indicated in the top-left part of the images. **(A, B)** 18 Mp images; **(C, D)** 15 Mp images.

Spike images were obtained in laboratory conditions using two protocols, “table” and “clip”, as described in the previous work (Genaev et al., 2019). For the “clip” protocol, images were obtained in four projections with 90° of rotation around the spike axis. The spikes on the table were imaged in the natural position at one image per spike. All images contained a ColorChecker Mini Classic target (https://xritephoto.com/camera) for scale determination. The resolution of the images was either 5,184 × 3,456 px (18 Mp) or 3,168 × 4,752 px (15 Mp). Examples of several typical spike images obtained using the “clip” protocol are shown in **Figure 2**.

A holdout dataset of 40 spike images of wheat hybrids obtained by the “clip” protocol supplemented the dataset for classification (see **Supplementary Table S2**). These images (as well as wheat accessions) were not used in the network training, validation, or testing. Spikes in 20 images from the holdout dataset had haired glumes, and 20 had hairless glumes. All preprocessing steps of the holdout dataset before classification were the same as for the main image dataset.

### 2.3 Image markup

To develop the automatic image segmentation model, a sample of 1,245 spike images for 249 plants was used, representing different accessions obtained using both “table” and “clip” protocols. This image dataset was described in a previous work in (Genaev et al., 2019). As a test sample, additional 30 images segmented manually were used. This dataset for testing is also identical to that from the previous work (Genaev et al., 2019). Initially, each image from the dataset was segmented automatically into four regions (background, ColorChecker target, awns, and spike body) using the program WERecognizer (Genaev et al., 2019). This program uses machine vision algorithms to segment spike images. Segmentation is performed into background and spike regions based on binarization in the HSV color space. After identification of the spike with its awns, the algorithm uses partial skeletonization of the spike image to extract the awns regions. For some images, the algorithm resulted in errors noticeable to the eye (violation of the integrity of the spikelet contour, misclassification of awns if they were densely spaced or touched together). Such images can be easily identified in the image preview mode and corrected manually in the raster graphic editor Gimp (https://www.gimp.org/). The fraction of images with correction was about 1%.

When labeling images by glume pubescence type, it was taken into account that glume hairiness is a classification trait at the level of varieties, which is characteristic of most wheat species (Goncharov, 2012; Yen et al., 2020). Therefore, the types of pubescence of each wheat species were used in labeling images. As an additional control, for a part of the images, glume pubescence was determined on the basis of visual analysis with a magnifying glass.

### 2.4 Image dataset stratification for segmentation and classification

For the segmentation task, the set of 1,245 images was randomly divided into training (70%) and validation (30%) parts. The test sample included 30 images as described in the previous section.

The dataset of spike images included plants of different species, images of the same spike in different protocols/projections, and was unbalanced by glume hairiness classes (there were *k* = 1.77 times more images of spikes with hairless glumes than haired ones). In addition, the glume hairiness trait is a characteristic of the plant belonging to a wheat species/variety (Dorofeev et al., 1979). Varieties within the same species are often very similar in glume hairiness. These factors had to be taken into account during image dataset stratification in order to maintain the proportions of different classes of images for the training, validation, and test subsamples.

Two methods of image dataset stratification were generated. These methods were aimed at keeping the proportion of the images in the training, validation, and testing subsamples as much as close to 80%:10%:10%, respectively. The first method, Str_TrainMaxDiversity, is to stratify the dataset by manual partitioning to obtain the maximum diversity of species represented in the training subsample. At the same time, the proportion *k* between the number of images with hairless glumes and the number of images with haired glumes is maintained only approximately in the validation/testing subsamples.

The second stratification method, Str_BalancedByClass, was aimed at minimizing the deviation of the *k* ratio in the three subsamples. For this purpose, the following steps of dataset stratification were implemented (**Supplementary Figure S3**):

(1) Images of a species presented in a fraction greater than 0.1 + *epsilon* of the total number of images were included in the training sample (lines 2–11).
(2) A species is randomly selected from the list of remaining species and a subsample type (training, test, or validation) (lines 40 and 41).
(3) If the selected subsample is training, then images of this type are added to it (lines 48–50).
(4) If the selected subsample is validation or testing and adding images of a species to it will result in the subsample fraction being less than the threshold (0.1 + epsilon), then images of this species are added to the subsample (lines 45–47). Otherwise, these images are added to the training sample (lines 49 and 50).
(5) If the fraction of testing and validation subsamples deviates from the target values within the epsilon value, all remaining species are added to the training subsample. The algorithm proceeds to step 6. Otherwise, steps 1–5 are repeated (lines 35–39).
(6) The maximum deviation of the parameter *k* between the three subsamples for the obtained partitioning, *dev*, is estimated (line 52).
(7) If *dev* is smaller than it was for the previous partitioning, this value and stratification (*dev_max*) are memorized, and *best_index* parameter is updated by the iteration index with the smallest *dev* value. Otherwise, the iteration counter is increased by 1, and current image split is added to the list of splits obtained previously. The algorithm proceeds to step 8 (lines 53–57).
(8) The algorithm is terminated if at least one of the following conditions is fulfilled: the number of iterations is more than 10,000; the *dev* value is less than 0.01. Otherwise, the iterating process is repeated from step 1 (line 28).

The proposed algorithm converged in less than 300 iterations, with a *dev_max* value of 0.009. The lists of species in the training, validation, and test samples for the two types of stratifications, class balance coefficients *k*, and the percentage of images in these samples are represented for both stratifications in **Supplementary Table S3**.

### 2.5 Image segmentation method

#### 2.5.1 Neural network model

The U-Net model (Ronneberger et al., 2015; Falk et al., 2019) was used for spike image segmentation. This model was developed for biomedical image analysis. The network contains two main parts: an encoder and a decoder. The encoder has a typical architecture of a CNN. The decoder part increases the dimensions of the feature maps, performs a deconvolution, which increases the number of feature maps, and combines them with the corresponding feature map from the compression part. The structure of the U-Net makes it possible to use different backbones represented by modern neural network models that have proven their effectiveness in image processing instead of the original version of the U-Net model (Konovalenko et al., 2022).

**Figure 3:**
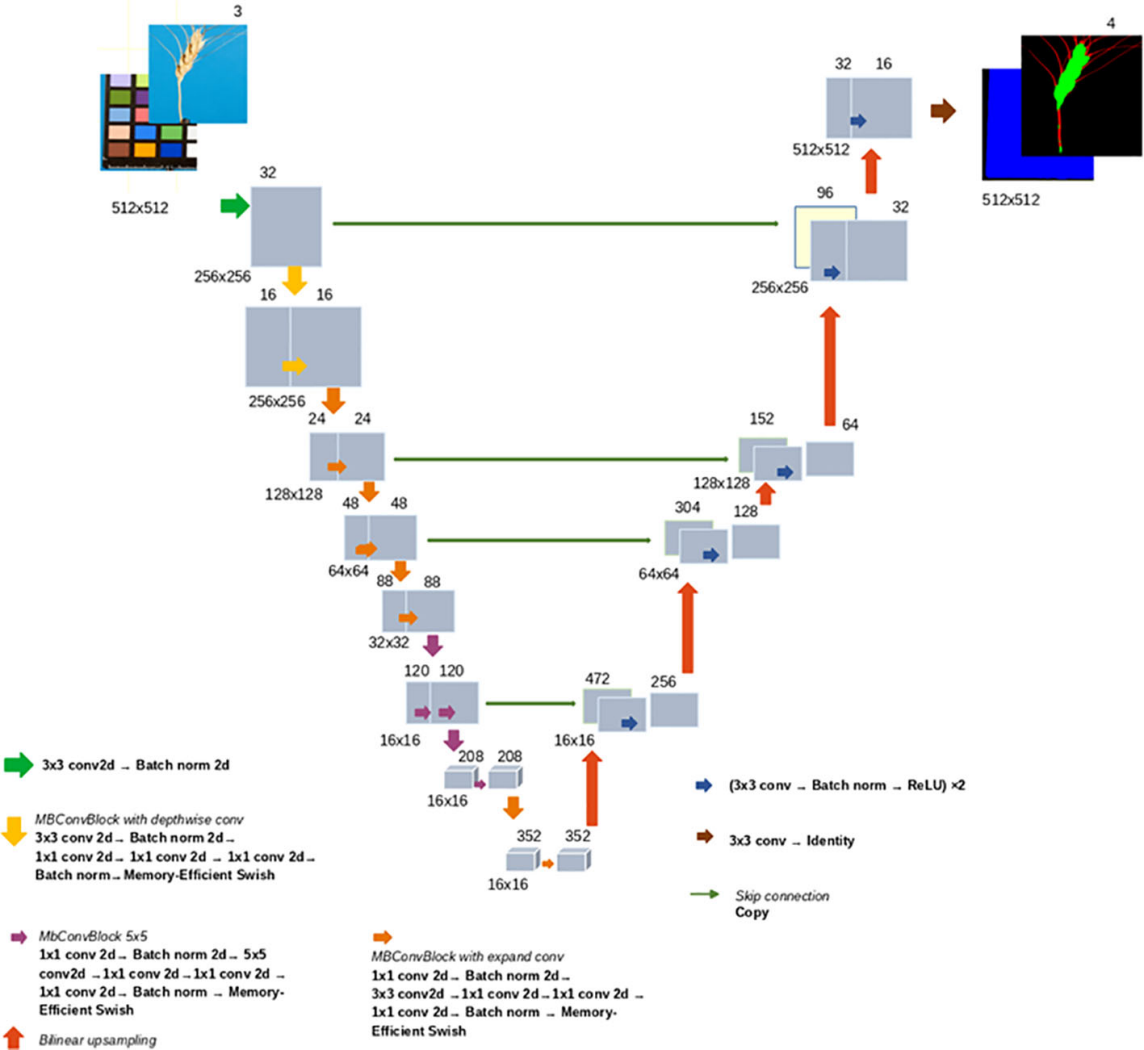
The U-Net architecture with the EfficientNet-B2 encoder used for the segmentation of spike images. The encoder part is shown on the left, and the decoder part is shown on the right. Gray rectangles indicate multichannel feature maps. The number of channels is given above the rectangles. The feature map size is indicated below the rectangles, on the left. Arrows indicate the type of operation and its direction.

Here the EfficientNet-B2 encoder was used in the U-Net network architecture. The EfficientNet network was proposed to solve the classification task (Tan and Le, 2019). It has a lightweight architecture based on AutoML. The main building block is the mobile inverted bottleneck (MBConv) (Sandler et al., 2018). Initially, a baseline network was developed, EfficientNet-B0. A family of topologies was obtained, differing by depth, width, and resolution of the network layers depending on the compound coefficient determining the total FLOPs for the network and varying from 0 (the most compact architecture) to 7 (the largest architecture) (Tan and Le, 2019). This architecture has also been successfully used as an encoder for segmentation tasks (Abedalla et al., 2021; Konovalenko et al., 2022; Jin et al., 2023). The images were segmented into four regions: background, ColorChecker target, spike body, and awns. The input data for the network were fragments of the original image of 512 × 512 pixels. For each pixel of the image, four weights from 0 to 1 were defined in the output of the network, according to the four specified classes. **Figure 3** shows the architecture of the U-Net network used for the segmentation of spike images with the EfficientNet-B2 encoder.

The EfficientNet-B2 includes seven types of sequential blocks built on the basis of the Conv and MBConv layers (Sandler et al., 2018; Tan and Le, 2019). In MBConv, the blocks consist of a layer that first expands and then compresses the channels, so direct connections are used between bottlenecks that connect much fewer channels than expansion layers. This architecture has in-depth separable convolutions that reduce calculation compared to traditional layers (Howard et al., 2017).

#### 2.5.2 Image pre-processing for segmentation and accuracy assessment

The preprocessing of images for segmentation consisted of the following steps:

(1) The image was split into 512 × 512 px tiles that overlapped each other in the 256 × 256 px area horizontally and/or vertically.

(2) An augmentation procedure was applied to the resulting tiles using the Albumentations library (Buslaev et al., 2020):

Transformations for the training subset:

 - HorizontalFlip (*p* = 0.5).

 - ShiftScaleRotate (*shift_limit* = 0.0, *scale_limit* = (0., 0.1), *rotate_limit* = 5, *interpolation* = cv2.INTER_LINEAR, *p* = 0.75).

 - ColorJitter (*brightness* = 0.4, *contrast* = 0.4, *saturation* = 0., *hue* = 0., *always_apply* = False, *p* = 0.75.).

 - Normalize.

Transformations for validation and test subsets:

 - Normalize.

(3) Tiles form the batches that the model receives as input.

Since tiles were obtained with overlap, one pixel in this analysis corresponded to the class prediction in several tiles. To obtain the final class prediction for a single pixel, the weight of each class was averaged over several overlapping tiles and the class with the maximum weight was selected as the predicted pixel class.

The IoU metric was used to evaluate the quality of class prediction (Everingham et al., 2010):


$$\text{IoU}=\frac{\left|A\cap^{\;}B\right|}{\left|A\cup^{\;}B\right|}=\frac{\left|A\cap^{\;}B\right|}{|\left|A\right|+\left|B\right|-\left|A\cap^{\;}B\right|},$$

where *A* denotes the pixels of the image region generated by segmentation using the segmentation algorithm and *B* denotes the manually marked pixels of the image region.

### 2.6 Image classification method

#### 2.6.1 Neural network models

**Figure 4:**
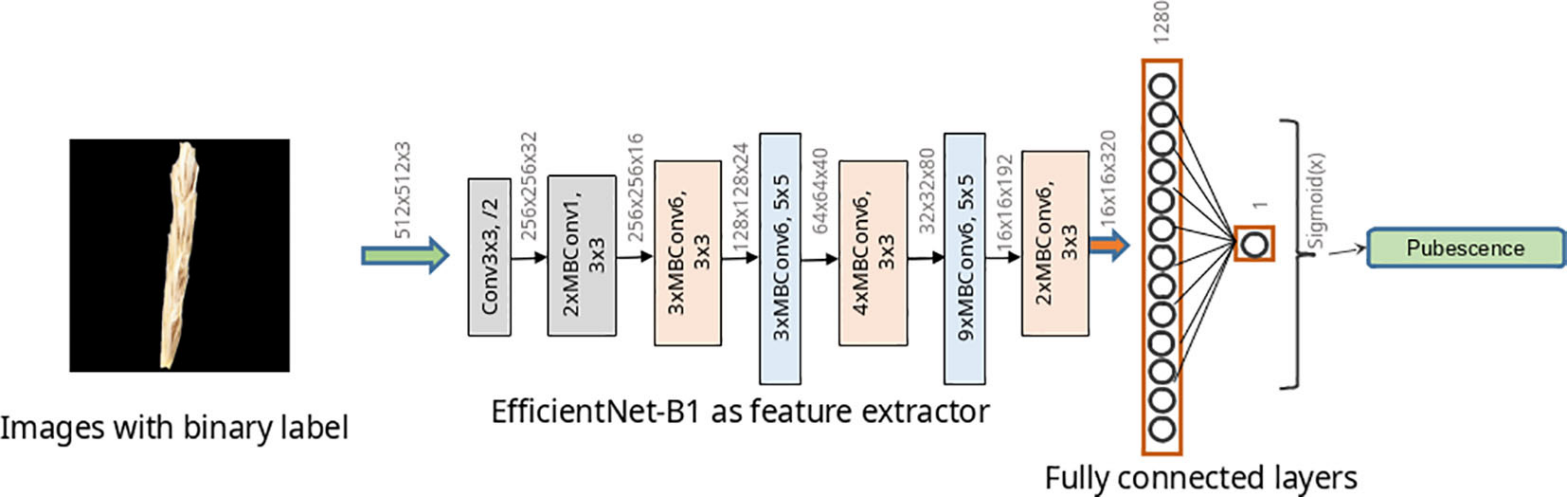
EfficientNet-B1 model architecture used to classify wheat spike images by glume pubescence. Rectangles indicate Conv and MBConv layers of various architectures.

To classify images according to the type of pubescence of glumes, ResNet-18 (He et al., 2016) and two networks of EfficientNet architecture (Tan and Le, 2019) were studied. The structure of the ResNet-18 neural networks is shown in **Supplementary Figure S1**. For the EfficientNet topology, two implementations were used: EfficientNet-B0 and EfficientNet-B1. The architecture of the EfficientNet-B1 network is shown in **Figure 4** and that of the EfficientNet-B0 network in **Supplementary Figure S2**. Estimates of the number of parameters and operations for these described networks are given in **Supplementary Table S4**. The abovementioned neural network architectures have previously demonstrated their effectiveness in solving plant image classification problems (De Camargo et al., 2021; Dourado-Filho and Calumby, 2021; Kanna et al., 2023).

The network models for image segmentation and classification were implemented using PyTorch v1.7.1 (Paszke et al., 2019). Initial weights for all models used in this work were obtained by training the network on the ImageNet dataset.

The Gradient-weighted Class Activation Map (Grad-CAM) algorithm from the TorchCAM package (https://github.com/frgfm/torch-cam) was used for the visualization of the network activation map. This technique assigns each neuron a relevance score for the class prediction in the output layer. GradCAM backpropagates this information to the last convolutional layer.

#### 2.6.2 Image preprocessing for classification and neural network parameters

**Figure 5:**
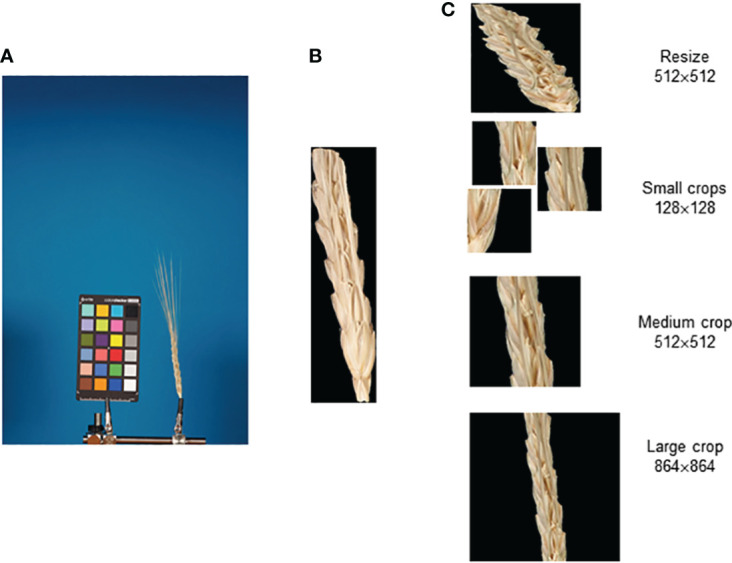
Main steps of the preparation of spike body image fragments for recognition of the glume pubescence. **(A)** Original spike image, **(B)** segmented spike body image in the bounding rectangle, and **(C)** images of the spike body or its fragments obtained as a result of resizing small, medium, and large crops.

The image preprocessing procedure for further classification is shown in **Figure 5**. The original image (**Figure 5A**) was segmented, and the spike body was extracted along with the bounding rectangle using the OpenCV library (Bradski and Kaehler, 2008). In this rectangle, the background pixels were assigned a black color (**Figure 5B**). Several methods were used to yield images of spikelet fragments of different sizes (**Figure 5C**): (1) resizing of the full bounding rectangle to 512 × 512 px, (2) small (128 × 128 px) crops containing random fragments of the spike body, (3) medium (512 × 512 px) crop of the spike central fragment, and (4) large (864 × 864 px) crop of the spike central fragment.

In the case of small crops, images in which the proportion of spike pixels was less than 30% were discarded. In case a medium or large crop exceeded the width of the bounding rectangle, pixels outside the bounding rectangle were assigned black color.

Input image preprocessing for the classification neural networks was implemented by randomly changing the brightness, saturation, and contrast using the Albumentations library (Buslaev et al., 2020).

The following parameters were used to train the classification neural networks: learning rate = 1e-7, weight decay = 1e-6, number of the epochs = 150, and training batch size = 16.

#### 2.6.3 Assessing the accuracy of spike classification

To assess the accuracy of the spike classification method on a sample of images, the authors compared the predicted class and its true value for each image and calculated the true positive (TP) values and true negative values (TN) as well as the total number of positive (pubescent, 1, *P*) and negative (glabrous, 0, *N*) values. Using these values, the accuracy was calculated for the set of images according to the formula: ACC = (TP + TN)/(*P* + *N*); the precision, PR = TP/(TP + FP); the F1 = 2·TP/(2·TP + FP + FN) (Alzubaidi et al., 2021); and the area under the curve (AUC) for the receiver operating characteristic (Huang and Ling, 2005).

The accuracy of glume hairiness detection was evaluated based on visual image analysis by an expert on a test dataset of the Str_TrainMaxDiversity stratification and in a holdout image dataset. In this case, no information about the spike belonging to a particular wheat variety was used. This analysis was performed to compare the accuracy of scale pubescence detection by eye and machine learning.

## 3 Results

### 3.1 Generating an image dataset

**Figure 6:**
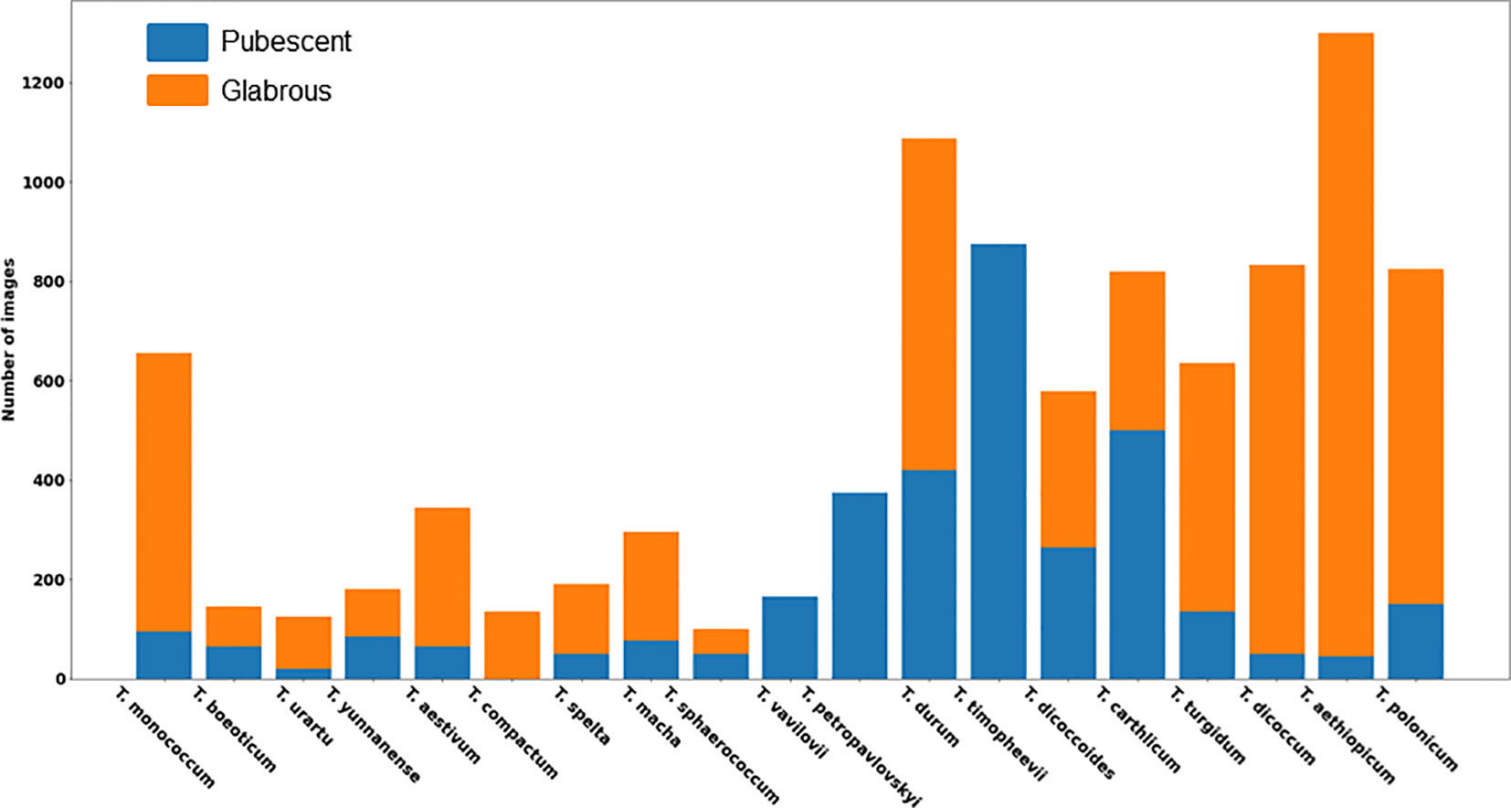
Distribution of the number of images of wheat spikes by species and glume pubescence.

A total of 9,679 spike images were obtained, including 3,499 with haired glumes and 6,180 with hairless glumes. The histogram of image distribution by species and by type of glume pubescence is presented in **Figure 6**.

The histogram shows that the distribution of spikes by glume pubescence differs significantly for different wheat species. There are species in which the samples are homogeneous in terms of glume pubescence: in *T. compactum* Host, all accessions in the sample have glabrous glumes, and in three species *T. timopheevii* (Zhuk.) Zhuk, *T. vavilovii* Jakubz, and *T. petropavlovskyi* (Udacz. et Migusch.) N.P. Gontsch., all accessions have pubescent glumes. There are species for which accessions with hairless glumes prevail: *T. monococcum* L., *T. urartu* Thum. ex Gandil, *T. aestivum* L., *T. spelta* L., *T. macha* Dek. et Men., *T. turgidum* L., *T. dicoccum* Schrank. ex Schublel, *T. aethiopicum* Jakubz., and *T. polonicum* L. There are species for which the ratio of accessions with pubescent and glabrous glumes does not differ much: *T. beoticum* Boiss., *T. yunnanense* (King ex S.L. Chen) N.P. Gontsch., *T. sphaerococcum* Perciv., *T. durum* Desf., *T. diccocoides* (Körn. ex Aschers. Et Graebn.) Schweinf., and *T. carthlicum* Nevski. Thus, the obtained data indicate that, even for a single species, accessions with both pubescent and glabrous glumes are quite frequently observed. The results obtained suggest taking into account both the species diversity of wheat accessions studied and their differences in the occurrence of pubescent/glabrous glumes during image dataset stratification.

### 3.2 Evaluation of the accuracy of image segmentation

The authors adapted and trained a U-Net architecture network with an EfficientNet-B2 encoder to segment images into the background, ColorChecker target, spike body, and awns. Estimates of the IoU parameter were obtained on a test sample of images. The ColorChecker target region is identified with the lowest error (IoU = 0.980), which can be explained by its simpler shape, close to a rectangle, with smooth edges. The spike body is identified with a lower but comparable accuracy (IoU = 0.947). The lower accuracy can be explained by the more complex shape of the spike and the presence of a large number of protrusions. For awns, the recognition performance is the lowest (IoU = 0.777). The awns occupy a smaller area in the image, which leads to the fact that errors will have a greater impact on the IoU score compared to the spike body and ColorChecker target. On the other hand, awns have a small thickness and a large boundary relative to the total area. The blurring of the boundary can introduce a significant uncertainty in the definition of awn contours.

Since the test images were the same as in the previous work (Genaev et al., 2019), we can directly compare the segmentation results. In the previous work, the authors obtained IoU (Jaccard’s coefficient) values for the spike body, 0.925, and for the awns, 0.660. This is lower by ~2.5% for the spike and by ~10% for the awns compared to the method from the present work.

**Figure 7:**
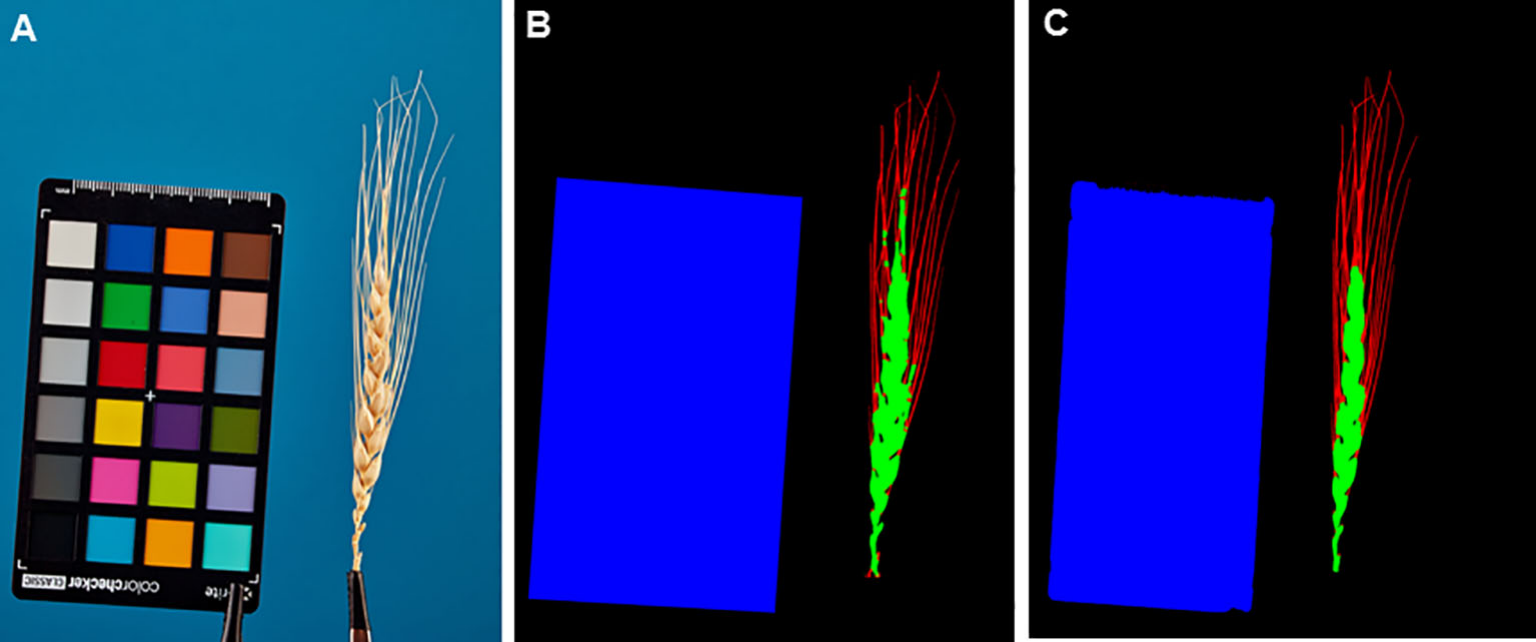
Example of spike image segmentation by the WERecognizer program and the U-Net model from the current work. **(A)** Initial spike image. **(B)** Image segmentation by the WERecognizer program. **(C)** Image segmentation by the U-Net. In **(B, C)**, the blue color represents the ColorChecker target, the green color represents the spike body, the red color represents awns, and the black color represents the background.

**Figure 7** shows examples of segmentation of one of the spike images performed using the WERecognizer program (Genaev et al., 2019) and using the U-Net model from this paper. The segmentation using the U-Net model yields smoother contours of the spike body compared to the WERecognizer method. The segmented regions are cohesive, while for the WERecognizer method, some of the pixels of the awns are marked as the spike body. At the same time, in the U-Net-segmented image (**Figure 7C**), one can notice the erosion of the ColorChecker target contour in the area where the ruler is placed on it. Other examples of segmented images are shown in **Supplementary Figure S3**. The results presented in these figures are consistent with general estimates of the accuracy of spike and awns recognition compared to WERecognizer. They demonstrate the higher accuracy of spike and especially awn image segmentation using the U-Net model.

### 3.3 Evaluating the accuracy of image classification

A preliminary analysis for the EfficientNet-B1 topology network using Str_TrainMaxDiversity training dataset stratification showed that the best accuracy on the test images was achieved using medium-sized spike body crops (**Supplementary Table S5**). Based on these results, input images of medium-sized crops were used in predicting the type of glume pubescence.

The authors trained and tested the accuracy of three neural network models on test and holdout datasets. The performance of the models on different stratification methods and on the holdout sample, respectively, are presented in **Table 1**. The last row of the table shows the performance estimates for recognizing glume pubescence by an expert.

**Table 1:** Evaluation of the performance of the EfficientNet-B0, EfficientNet-B1, and ResNet18 models and expert classification using test and holdout image datasets. Best performance metrics for each stratification method shown in bold.

Stratification method	Model	Test dataset				Holdout dataset			
		ACC	PR	F1	AUC	ACC	PR	F1	AUC
Str_BalancedByClass	EfficientNet-B0	0.79	**0.93**	0.60	0.87	0.63	0.78	0.55	0.76
	EfficientNet-B1	**0.89**	0.81	**0.85**	**0.96**	**0.83**	**0.78**	**0.84**	**0.89**
	ResNet18	0.87	0.88	0.81	0.92	0.73	0.77	0.55	0.77
Str_TrainMaxDiversity	EfficientNet-B0	0.73	0.61	0.30	0.72	0.75	**0.86**	0.71	0.78
	EfficientNet-B1	**0.85**	**0.75**	**0.74**	**0.86**	**0.85**	0.82	**0.84**	0.86
	ResNet18	0.79	0.63	0.65	0.82	0.83	0.84	0.74	**0.87**
	Expert by an eye	0.92	1.00	0.95	–	0.80	0.88	0.78	–

The results presented in the table demonstrate that, for Str_BalancedByClass stratification (balanced by classes), the accuracy estimates on the test sample are slightly higher than for Str_TrainMaxDiversity stratification. At the same time, for the holdout dataset, no noticeable differences were observed for the different training samples. Among the models, the EfficientNet-B1 model demonstrates the highest accuracy. Its advantage is observed both for different stratification methods and for both subsamples. At the same time, the performance estimates for this network on the holdout dataset when training on data stratified by different methods differ only slightly.

The results of the performance estimation for recognizing pubescence by an expert show that, on the test dataset (Str_TrainMaxDiversity), the expert classification performance (F1 = 0.95) is noticeably higher than for the best neural network model (0.74). On the holdout dataset, the results are quite comparable: F1 for the expert and the best model are 0.78 and 0.84, respectively.

**Figure 8:**
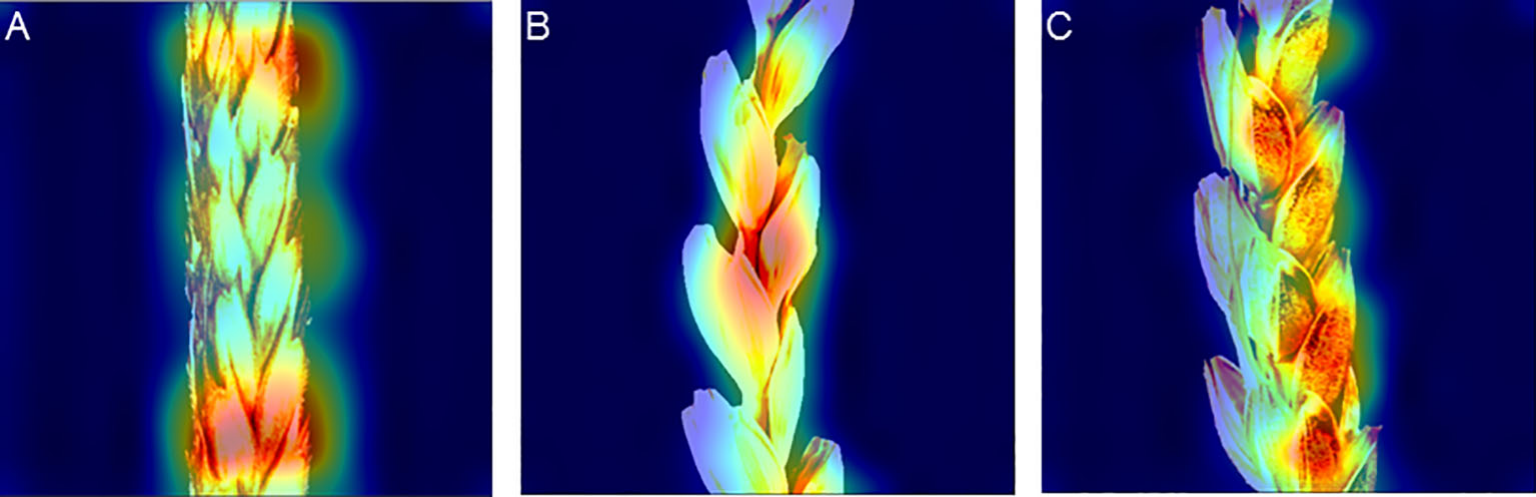
Activation maps of the EfficientNet-B1 model for classifying spike images by glume pubescence evaluated on the image dataset with Str_BalancedByClass stratification. **(A)** Activation regions are located at the boundary between spike and background (top) and at the edges of glumes in the central part of the spike body. **(B)** Activation regions are located at the edges of glumes in the central part of the spike. **(C)** Activation regions are located in the central part of glumes.

Examples of the activation map for the spike image fragments are shown in **Figure 8**. The figure demonstrates that the network focuses either at the edges of glumes or at the border between the spike body and background (**Figure 8A**), in the central part of the spike image (**Figures 8A, B**), or at the central part of glumes (**Figure 8C**). The location of the activation regions on the glume edges is well explained: the hairs at the edges are most clearly visible in the image.

### 3.4 Effect of image magnification and distortion on classification performance

**Figure 9:**
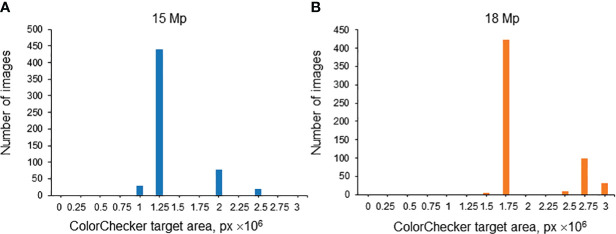
Histogram of relative areas of the ColorChecker target in the images of the test subsample (Str_TrainMaxDiversity stratification method) for 15 Mp **(A)** and 18 Mp **(B)** images. The X-axis is the ColorChecker target area size, in px. The Y-axis is the number of images.

The spike images obtained in this work vary in magnification due to different distances between the camera and the spike in various series of images. This distance was the same in one series of images. Since the size of the ColorChecker target was the same, the magnification can be characterized by its area expressed in pixels. It is, however, necessary to take into account that, in terms of resolution, the images were of two types: 15 Mp and 18 Mp. Therefore, for each resolution, the distribution of images was plotted by the ColorChecker target area expressed in pixels. The results are summarized in **Figure 9**. A threshold of 2.25 × 10^6^ px was chosen for the ColorChecker target area: image magnification was considered large if the ColorChecker target area was larger than this threshold and small if smaller.

The accuracy estimates of the EfficientNet-B1 model trained on the dataset obtained by the Str_TrainMaxDiversity stratification method were calculated.

It was found that, for images of higher magnification, the performance estimates were ACC = 0.81, PR = 0.70, and AUC = 0.91. For images of smaller magnification, the values of accuracy parameters were ACC = 0.80, PR = 0.55, and AUC = 0.80. The difference in the AUC parameter was 10% in favor of the higher-magnification images. However, it is important to note that the ratio of images with pubescent glumes to those with glabrous glumes differed in these two sets. Specifically, it was 0.14 for the images with large magnification and 0.86 for the images with small magnification. This discrepancy might also influence the performance of glume pubescence recognition in these two subsamples.

The results show that imaging conditions affect the accuracy of pubescence detection in the images. This issue was investigated in more detail. For this purpose, a sample of 35 images of the *T. beoticum*/321-329 accession (hairless glumes) and 46 images of the *T. vavilovii*/271-280 accession (haired glumes) was generated from the Str_TrainMaxDiversity stratification test sample. The EfficientNet-B1 model for these images classified the type of pubescence without error. Using the Albumentations library, blurring was applied to these images with kernel sizes of 3, 4, 5, and 6 pixels. After each transformation, the method was applied to recognize the pubescence of scales, and its accuracy was evaluated. The results are summarized in **Table 2**. It can be seen from the table that the accuracy of glume pubescence recognition decreases as the distortion increases, but it is still quite high for the kernel size of 3 and 4 pixels. It can be assumed that a slight blurring of the spike image (due to its deviation from the lens focus) does not significantly affect the accuracy of pubescence recognition. It should also be noted that the errors in this experiment were solely due to the misclassification of spikes with pubescent glumes.

**Table 2:** Effect of image blurring on the accuracy of ear classification by scale pubescence.

Blur kernel size, px	ACC	PR	AUC
3	0.86	1	0.99
4	0.58	1	0.96
5	0.46	1	0.86
6	0.46	1	0.73

The first column shows the size of the blurring kernel, and the following columns show estimates of the pubescence identification accuracy.

Additionally, the effect of changing the image brightness on the accuracy of spike classification was investigated. For this purpose, the RGB channel intensities for each pixel were changed upward and downward by 20%, 40%, and 60% for the abovementioned set of images. The classification accuracy was evaluated for the distorted images. The results are summarized in **Table 3**. The results appear similar: the greater the image distortion, the lower the accuracy of the method (due to errors in classifying ears with haired glumes). Note, however, that the decrease in brightness affects the accuracy to a lesser extent than its increase.

**Table 3:** Effect of image brightness on the accuracy of ear classification by scale pubescence.

R, G, B channel intensity change, %	ACC	PR	AUC
+20	0.91	1	0.97
+40	0.67	1	0.94
+60	0.46	1	0.84
-20	0.93	1	0.99
-40	0.83	1	0.99
-60	0.58	1	0.99

The first column shows the value of the relative change in the intensity of the R, G, B channels for the image pixels.

## 4 Discussion

This paper proposes an integrated method to assess glume pubescence in images of wheat spikes obtained under laboratory conditions. It is suggested that this image format may be typical when digitizing genetic collections for further phenotyping (Fu, 2017; Nguyen and Norton, 2020). The images (**Figure 2**) include the entire spike body, awns that can occupy a fairly large area, and a ColorChecker target for scaling and color normalization. For the optimal processing of a large number of images, the camera and the spike are at a constant distance in the image series, regardless of the size of the spikelet. This leads, however, to the fact that, for small-sized spikes, the quality of its representation in the image may decrease. Note that obtaining more detailed images required varying the distance from the camera to the spike depending on the size of its body and awns. However, this would significantly slow down the imaging process. The analysis showed that, for these images, it was possible to determine the pubescence of the glumes with good accuracy, even without using a magnification that allowed obtaining images containing fine details of the glume hairs. Nevertheless, the analysis of the accuracy of pubescence detection for images at different magnifications showed (10% difference in AUC parameter) that this was an important factor affecting the accuracy of the method. A similar conclusion was obtained earlier in the spike segmentation task (Genaev et al., 2019): the accuracy of spike body and awn region identification was higher for images with higher magnification. Note that the analysis of activation maps demonstrates that the most informative regions in the image are those in which hairs are most distinguishable (edges and central regions of scales).

The first step of the proposed method was to identify the spike body in the image in order to use only its contour for classification. This segmentation step was chosen to exclude irrelevant regions in the image, such as the ColorChecker target, awns, and background. For this purpose, a network based on the U-Net topology was used. The results demonstrated high segmentation accuracy. It is comparable to the performance of other deep learning methods—for example, Ullah et al. (2021) segmented spikes in the image with the IoU parameter from 0.610 to 0.922 depending on the segmentation algorithm, with higher values for CNN methods. Note that the segmentation based on the neural network developed in this work can also be used also for spike morphometry.

When training the neural network, two stratifications were used for image sampling. They differed in the ratio of samples with haired and hairless glumes in the training/validation/test subsamples from 1.574 to 1.98. Note that this ratio can vary greatly in samples of different wheat accessions—for example, in Börner et al. (2005), the proportion of *T. aestivum* accessions with haired glumes varied quite strongly among populations with different geographical origins—for example, in accessions from Poland, Kenya, Tunisia, and some others, haired glumes were not represented at all. In accessions from Turkey, Cyprus, and India, their fraction reached ~30%. In accessions from Libya, their fraction was at a maximum, 65%. On average, the proportion of samples with pubescent glumes for bread wheat representatives was 13%. In the dataset considered in this study, the fraction of accessions with pubescent glumes is generally higher (from ~40% to 30%). However, the considered sample contains different wheat species. Note that for bread wheat *T. aestivum* in our dataset (**Figure 6**), this ratio is consistent with the data of Börner et al. (2005).

The conducted analysis demonstrated that the best network performance was observed for the EfficientNet-B1 architecture, which is probably due to the optimal ratio between the number of network parameters and the size of training data in the considered case. For data in which all three types of subsamples are balanced equally in terms of the proportion of spikes with pubescent glumes (Str_BalancedByClass stratification method), the accuracy is maximized (F1 = 0.85, AUC = 0.96). At the same time, for the holdout sample, the differences in which stratification was used for training are small. The obtained estimates are typical in solving image-based plant classification problems—for example, Rolland et al. (2022) classified cotton images by the intensity of pubescence (nine classes) using the ResNet model and obtained 84.85% for the accuracy of all images and 91.36% for leaf accuracy, which were close to the estimates of the present study (**Table 1**). The obtained estimates are typical in solving image-based plant classification problems.

The most common classification of plants into varieties and landraces is based on image analysis of grains. In the work of Landa et al. (2021), grape seeds were classified as belonging to a certain variety on the basis of image analysis and LDA. Depending on the type of stratification, the accuracy varied from 79% to 93%. Artificial neural networks were used to classify grains into bread or durum wheat based on 21 features obtained from the image analysis (Sabanci et al., 2020). The classification accuracy was about 99%. The CNN model of wheat grain image classification into 15 varieties was used in Lingwal et al. (2021) and yielded an accuracy of 0.97 on the test dataset.

The proposed method for analyzing spike images operates relatively quickly. A computer with a GPU Nvidia RTX 2080ti was used to train and evaluate CNN models. On average, processing one image to predict glume pubescence took 50 s, with segmentation taking up 25 s of this time. Thus, the proposed method offers rapid and accurate phenotyping of wheat genetic collection images, enabling the characterization of glume pubescence trait diversity.

## 5 Conclusion

An integrated method based on the use of CNN to classify wheat spike images by glume pubescence was proposed. The method includes two stages of analysis: image segmentation based on the U-Net network with an EfficientNet-B2 encoder and classification of spike body images by glume pubescence (pubescent or glabrous). The proposed approach allows distinguishing spikes in the image with high accuracy and can be used in various downstream spike analyses.

The classification of images based on glume pubescence achieved the highest performance using the network featuring the EfficientNet-B1 architecture. This network proved highly effective when trained on both balanced and unbalanced image datasets, demonstrating results comparable to those obtained via expert classification. The analysis underscores the effectiveness of deep machine learning networks in extracting crucial classification features from spikes, thereby establishing the utility of these methods in large-scale phenotyping of wheat genetic collections.

## Data availability statement

The original contributions presented in the study are included in the article/**Supplementary Material**. Further inquiries can be directed to the corresponding author.

## Author contributions

NA: Formal analysis, Investigation, Methodology, Software, Validation, Visualization, Writing – original draft. MG: Investigation, Software, Conceptualization, Formal analysis, Methodology, Project administration, Supervision, Validation, Writing – original draft. RE: Investigation, Software, Writing – original draft. EK: Data curation, Investigation, Validation, Writing – original draft. YK: Data curation, Resources, Writing – original draft. VK: Data curation, Methodology, Resources, Writing – original draft. NG: Conceptualization, Data curation, Writing – review & editing. DA: Conceptualization, Funding acquisition, Methodology, Project administration, Resources, Supervision, Validation, Writing – original draft, Writing – review & editing.
