# Supplementary material for: Image-based classification of wheat spikes by glume pubescence using convolutional neural networks
Source: Front Plant Sci. 2024 Jan 12;14:1336192. doi: 10.3389/fpls.2023.1336192 (PMC10811101; doi:10.3389/fpls.2023.1336192)
Supplement: Supplementary file 1 [file DataSheet_1.docx]

Supplementary Material

# Supplementary Figures and Tables

## Supplementary Tables

**Table S1.** List of *Triticum* species from N.P. Goncharov collection used for analysis and classification of spikes by glume hairiness and the corresponding number of images.

| # | Species | Number of accessions | Number of images | |
| --- | --- | --- | --- | --- |
|  |  |  | haired glume | hairless glume |
| Diploid species (2*n* = 2*х* = 14) | | | | |
| 1 | *T. monococcum* L. | 18 | 95 | 560 |
| 2 | *T. boeoticum* Boiss. | 7 | 65 | 80 |
| 3 | *T. urartu* Thum. ex Gandil. | 10 | 20 | 105 |
| Tetraploid species (2*n* = 4*х* = 28) | | | | |
| 4 | *T. durum* Desf. | 24 | 420 | 667 |
| 5 | *T. timopheevii* (Zhuk.) Zhuk. | 20 | 875 | 0 |
| 6 | *T. dicoccoides* (Körn. ex Aschers. et Graebn.) Schweinf. | 18 | 264 | 315 |
| 7 | *T. carthlicum* Nevski | 20 | 500 | 320 |
| 8 | *T. turgidum* L. | 14 | 135 | 500 |
| 9 | *T. dicoccum* (Schrank) Schuebl. | 19 | 50 | 783 |
| 10 | *T. aethiopicum* Jakubz. | 30 | 45 | 1255 |
| 11 | *T. polonicum* L. | 17 | 150 | 675 |
| Hexaploid species (2n = 6х = 42) | | | | |
| 12 | *T. yunnanense* (King ex S.L. Chen) N.P. Gontsch. | 4 | 85 | 95 |
| 13 | *T. aestivum* L. | 8 | 65 | 280 |
| 14 | *T. compactum* Host | 3 | 0 | 135 |
| 15 | *T. spelta* L. | 4 | 50 | 140 |
| 16 | *T. macha* Dek. et Men. | 8 | 76 | 220 |
| 17 | *T. sphaerococcum* Perciv. | 2 | 50 | 50 |
| 18 | *T. vavilovii* Jakubz. | 5 | 180 | 0 |
| 19 | *T. petropavlovsky* Udacz. et Migusch. | 8 | 374 | 0 |
|  | Total | 239 | 3499 | 6180 |

**Table S2.** List of wheat hybrids from N.P. Goncharov collection used for testing the performance of the classification of spikes by glume hairiness as holdout dataset.

| № | Hybrid combination |
| --- | --- |
| **F_1_ hybrids** | |
| Vegetation II-20 (sowing in February, 2020) | |
| 7762 | *T. durum* x *T. dicoccoides* |
| 7785 | *T. durum* x *T. polonicum* |
| 7807 | *T. aethiopicum* x *T. timopheevii* |
| 7818 | *T. aethiopicum* x *T. carthlicum* |
| 7839 | *T. dicoccum* x *T. carthlicum* |
| 7849 | *T. timopheevii* x *T. polonicum* |
| 7861 | *T. timopheevii* x *T. carthlicum* |
| 7889 | *T. carthlicum* x *T. dicoccum* |
| 7895 | *T. polonicum* x *T. aethiopicum* |
| 7901 | *T. polonicum* x *T. dicoccum* |
| Vegetation X-20 (sowing in October 2020) | |
| 6802 | *T. dicoccoides* x *T. timopheevii* |
| 6805 | *T. durum* x *T. aethiopicum* |
| 6812 | *T. durum* x *T. dicoccum* |
| 6820 | *T. durum* x *T. carthlicum* |
| 6831 | *T. aethiopicum* x *T. dicoccoides* |
| 6842 | *T. aethiopicum* x *T. durum* |
| 6850 | *T. aethiopicum* x *T. durum* |
| 6861 | *T. aethiopicum* x *T. polonicum* |
| 6873 | *T. dicoccum* x *T. durum* |
| 6892 | *T. polonicum* x *T. dicoccoides* |
| Vegetation II-21 (sowing in February 2021) | |
| 6723 | *T. timopheevii* x *T. dicoccum* |
| 6729 | *T. timopheevii* x *T. dicoccum* |
| 6732 | *T. dicoccum* x *T. dicoccoides* |
| 6747 | *T. timopheevii* x *T. durum* |
| 6765 | *T. carthlicum* x *T. aethiopicum* |
| Vegetation X-21 (sowing in 2021) | |
| 6725 | *T. durum* x *T. aethiopicum* |
| 6726 | *T. aethiopicum* x *T. durum* |
| 6735 | *T. dicoccum* x *T. dicoccoides* |
| 6763 | *T. polonicum* x *T. carthlicum* |
| 6778 | *T. carthlicum* x *T. timopheevii* |
| 6801 | *T. durum* x *T. turgidum* |
| **F_2_ hybrids** | |
| Vegetation X-20 (sowing in October 2020) | |
| 7382 | *T. durum* x *T. dicoccoides* |
| 7384 | *T. durum* x *T. dicoccoides* |
| Vegetation V-21 (sowing in May 2021) | |
| 16n1 | *T. dicoccum* x *T. aethiopicum* |
| 28n3 | *T. dicoccum* x *T. durum* |
| 28n4 | *T. dicoccum* x *T. durum* |
| 2n2 | *T. durum* x *T. dicoccoides* |
| 3n11 | *T. durum* x *T. dicoccoides* |
| Vegetation in X-21 (sowing in October 2021) | |
| 7024 | *T. durum* x *T. dicoccum* |
| 7044 | *T. durum* x *T. dicoccum* |

**Table S3.** Wheat species representation in two datasets obtained as result of different stratification methods: representing maximal species diversity in training sample (Str_TrainMaxDiversity) and minimal deviations between the ratio of haired/hairless glume accessions in train/validation/test samples (class balance, Str_BalancedByClass).

| Subsample | Species | Class balance coefficient | Fraction of images |
| --- | --- | --- | --- |
| Stratification: Str_TrainMaxDiversity | | | |
| Training | *T. aestivum*  *T. aethiopicum*  *T. carthlicum*  *T. compactum*  *T. dicoccum*  *T. durum*  *T. monococcum*  *T. petropavlovsky*  *T. spelta*  *T. sphaerococcum*  *T. timopheevii*  *T. turgidum*  *T. urartu*  *T. yunnanense* | 1.769 | 79.2 % |
| Validation | *T. dicoccoides*  *T. macha* | 1.574 | 9 % |
| Testing | *T. beoticum*  *T. polonicum*  *T. vavilovii* | 1.987 | 11.8 % |
| Stratification: Str_BalancedByClass | | | |
| Training | *T. aethiopicum*  *T. boeoticum*  *T. carthlicum*  *T. dicoccum*  *T. durum*  *T. monococcum*  *T. petropavlovsky*  *T. polonicum*  *T. sphaerococcum*  *T. timopheevii*  *T. turgidum* | 1.772 | 79.2% |
| Validation | *T. dicoccoides*  *T. macha*  *T. urartu* | 1.777 | 10.3% |
| Testing | *T. aestivum*  *T. compactum*  *T. spelta*  *T. vavilovii*  *T. yunnanense* | 1.781 | 10.5% |

**Table S4.** Characteristics of the neural networks used for the classification of the wheat spike images.

| Network model | Number of parameters | FLOPs |
| --- | --- | --- |
| ResNet18 | 1.17e+7 | 1.82e+9 |
| EfficientNet-B0 | 5.3e+6 | 0.39e+9 |
| EfficientNet-B1 | 7.8e+6 | 0.70e+9 |
| EfficientNet-B2 | 9.2e+6 | 1.0e+9 |

**Table S5.** The influence of the type of input images on the accuracy of the classification of ears by the type of scale pubescence for the network topology EfficientNet-B1.

| Input image type | Validation dataset | | | Test dataset | | |
| --- | --- | --- | --- | --- | --- | --- |
|  | ACC | PR | AUC | ACC | PR | AUC |
| Resize | 0.71 | 0.64 | 0.77 | 0.69 | 0.49 | 0.71 |
| Small crops | 0.79 | 0.66 | 0.90 | 0.72 | 0.51 | 0.76 |
| Medium crops | **0.95** | **0.93** | **0.98** | **0.85** | 0.75 | **0.86** |
| Large crops | 0.87 | 0.92 | 0.95 | 0.77 | **0.80** | 0.81 |

## Supplementary Figures


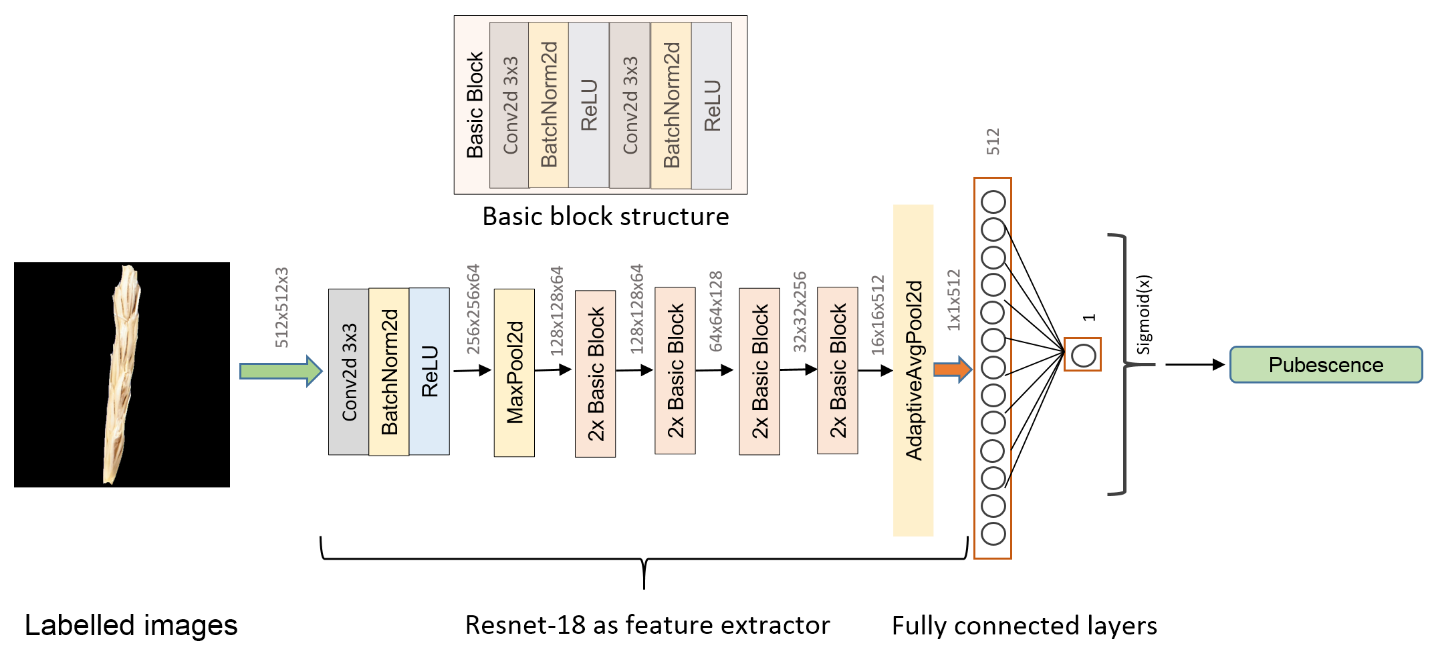


**Figure S1.** Architecture of the Resnet-18 neural network for classification of wheat spike images by glume pubescence.

**
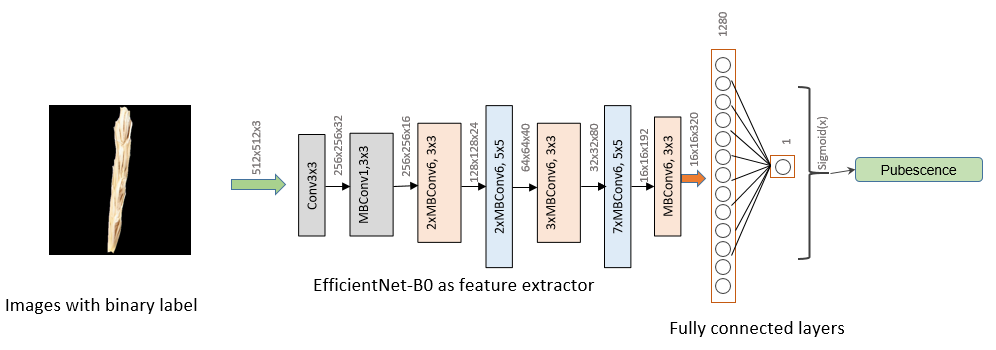
**

**Figure S2.** Architecture of the EfficientNet-B0 neural network for classification of wheat spike images by glume pubescence.


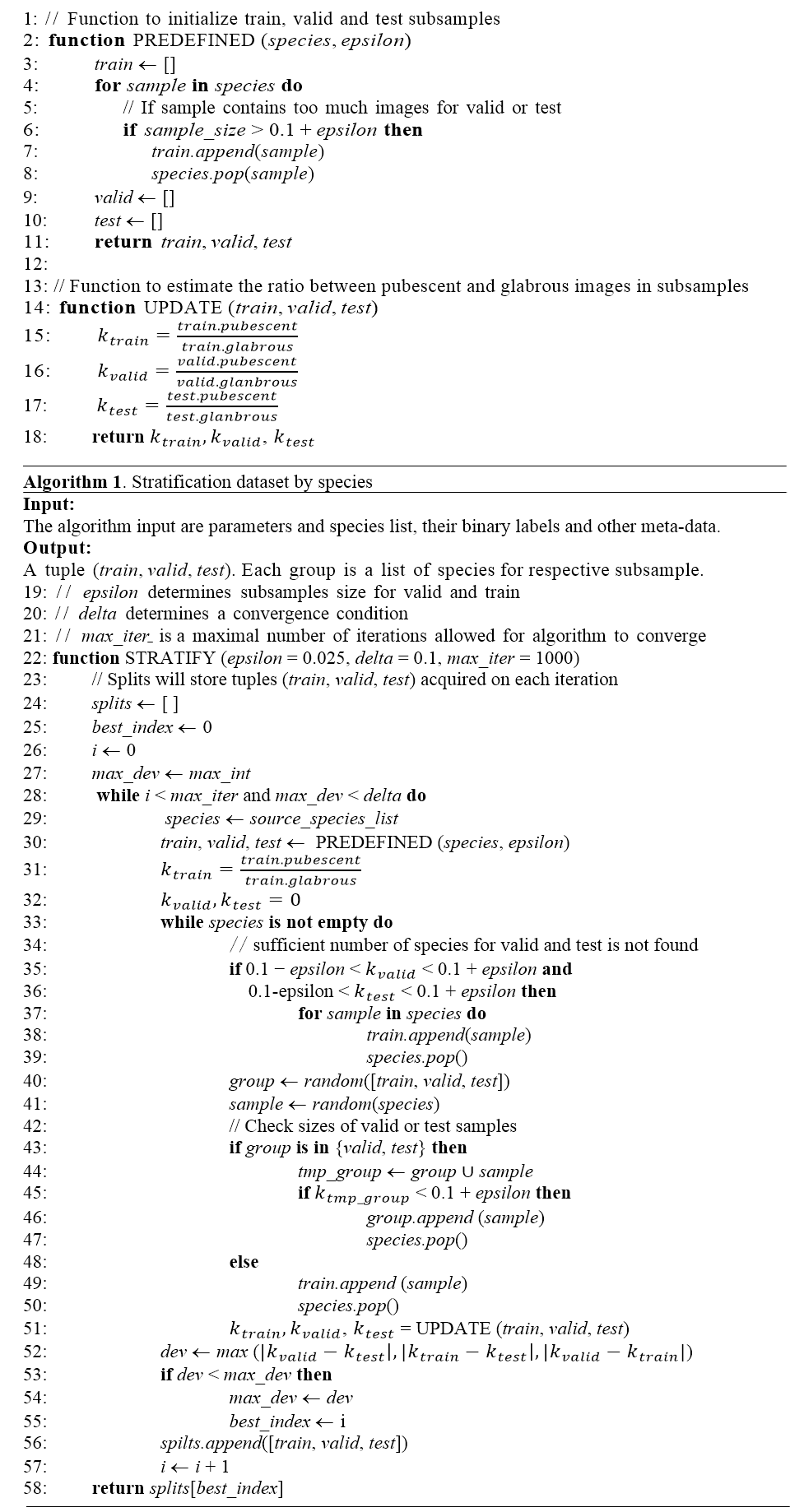


**Figure S3.** Algorithm for image sample stratification by species aimed at minimizing the deviation of the *k* ratio between pubescent and glabrous accessions in the training, validation and test datasets.


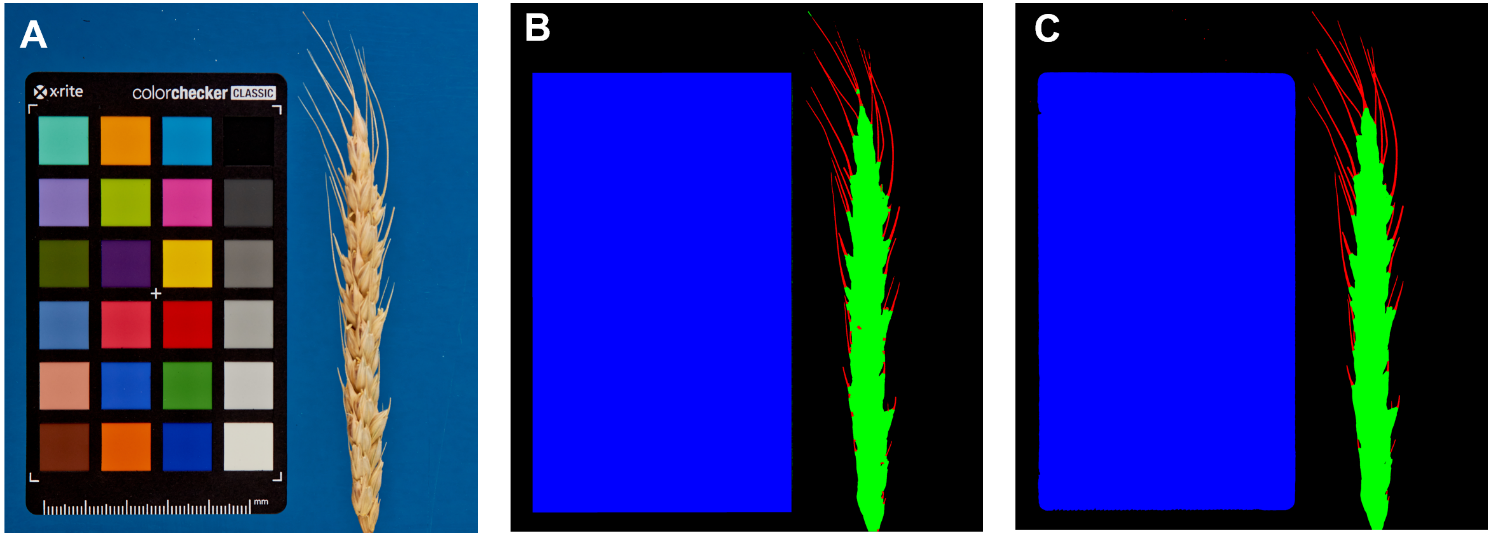


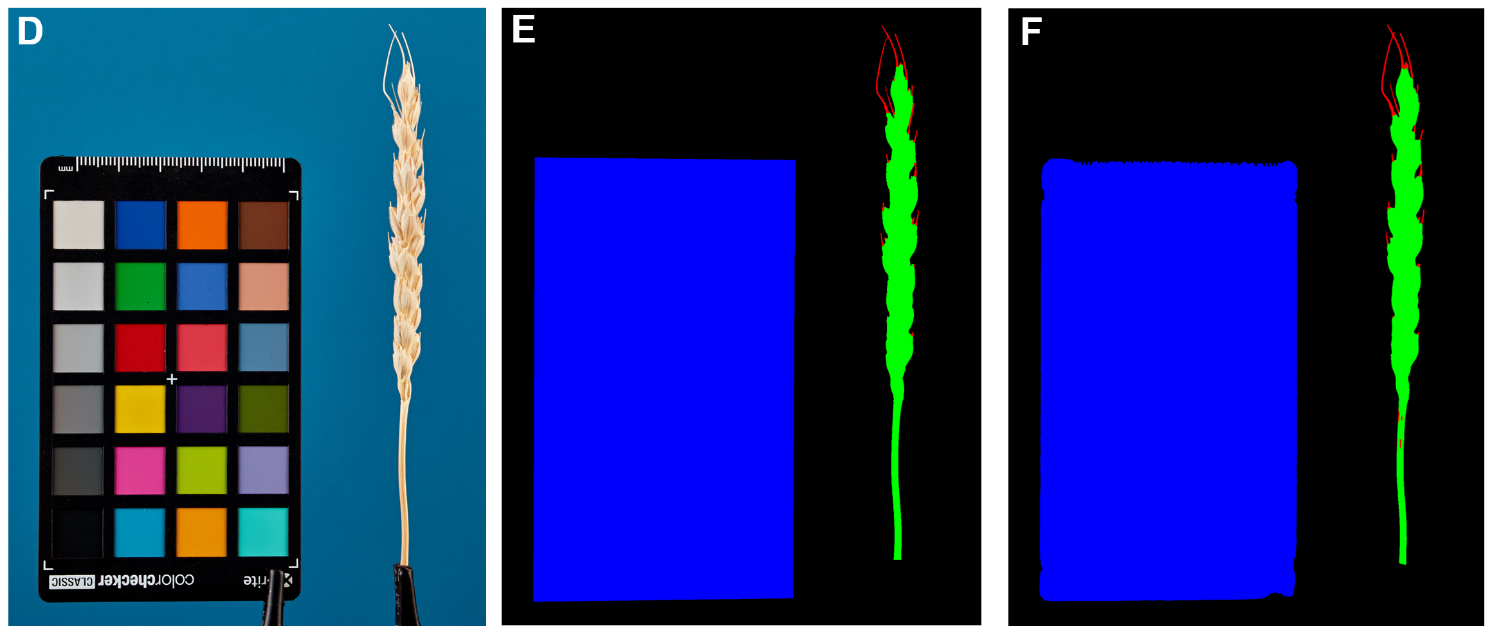


**Figure S4.** Example of spike image segmentation by the WERecognizer program and the U-Net model from the current work. (A,D) Initial spike image; (B,E) image segmentation by the WERecognizer program; (C,D) image segmentation by the U-Net. In (B,C,E,F) panels, the blue color represents the ColorChecker target, the green color represents the spike body, the red color represents awns, and the black color represents the background.
